# Keeping the journal on its track, but with new guidelines

**DOI:** 10.1016/S1808-8694(15)30025-2

**Published:** 2015-10-19

**Authors:** João Ferreira de Mello Júnior

Dear colleagues,

I was greatly honored to be invited to be the Editor of the Brazilian Association of Otolaryngology – Cervico-Facial Surgery Journal. In any medical journal, such office requires great responsibility. Specially for me this demand will be even greater, because the teams that preceded me were outstanding, they were even able to index our journal internationally, in both Scielo and Medline, being considered Qualis national A and Qualis international C by CAPES.

The quality achieved by our publication was only possible thanks to the effort of the colleagues who have submitted their scientific research all these years. My main goal is to keep our journal on its current track, and try to raise the standard we have reached so far. This will only be possible if we keep our scientific level, because only then our papers and journal will be quoted in other authors’ references. The more quotes we have in other publications, the more important our journal becomes. Thus, we may aim at achieving Qualis international B or class A. This objective will not be met overnight, requiring time and work, from the authors, editors, associates, editorial body and the whole journal team.

As the Editor, and being part of a team coordinated by Prof. Sílvio Caldas Neto (Publications Director) and Prof. Regina H. G. Martins (Assistant Director of Publications), some points regarding our work have to be touched upon.

Many colleagues seek us out to know why it takes so long to publish some of their papers, specially “case reports”. We have received a number of these papers, besides those already reviewed and awaiting publication. Unfortunately we can not publish as many of those as we wished, because in order to keep the classification we’ve reached we have to give priority to original papers. The more original work published, the greater is the chance of being quoted.

In an attempt to mitigate the problem, soon the authors of this type of articles, already submitted to the journal will be questioned as to the feasibility of changing their current format. Our major goal is to make them more objective and clear for the readers. Those who choose to re-format them will have their papers published faster, because they will be printed in special issues. Those who wish to keep their case reports in the original format will have to be more patient because the number of such papers per journal issue is limited. The new format guidelines are defined and available in the files receiving system - through the Internet. Besides, in a temporary basis, new case reports are not being accepted, however, as soon as we can accepted again, they have to follow the new guidelines. At the journal discretion, special cases may follow other formats.

Still, about the delay in reviewing and publishing papers, some colleagues that have submitted their papers now for some while, have contacted us to enquire the whereabouts of their work. We are now implementing changes in the review process in an attempt to expedite the whole process.

Another important issue is the re-registration of our reviewers. Otolaryngology is a broad medical field, encompassing allergy, audiology, head and neck surgery, skull-maxilla-facial, facial plastic surgery, swallowing, stomatology, pharyngology, laryngology, voice, otoneurology, pediatric ENT, otology, rhinology, sleep disorders, etc. We have recently asked the reviewers to chose the areas they would most like to work with, so as to have the most qualified personnel to review the papers.

As to maintaining the scientific quality of our journal, we reinforce to those colleagues who wish to publish their work involving human beings or animals, that these papers should be submitted together with the approval protocol number from the Ethics Committee where the investigation was carried out.

Our journal is available to all those who wish to express their opinions about the papers published. Thus, following the suggestion of some colleagues, we are opening a new session called “Letter to the Editor”, where readers may express their opinions about some paper or theme published. However, in order to guarantee a right of response, this letter will be referred to the author of the commented upon paper, before it is published. If the author wishes to, his/her response will be published together with the letter. The guidelines for these texts are available at the journal internet site.

And finally, these first lines I write as the Editor are part of a very special journal issue. In this issue we have published some of the award winning scientific work presented at the last Triological Society Meeting. Everyone will be able to appreciate the scientific quality of our colleagues, encompassing different fields of otolaryngology.

I thank all of you who trusted me with this office.

Best regards

